# Divergent sucker-corm endophytic microbiota underpins the progressive decline of Fusarium-wilt incidence in resistant bananas across ratoon cycles

**DOI:** 10.3389/fmicb.2026.1676292

**Published:** 2026-02-03

**Authors:** Sainan Xiang, Dandan Tian, Jiahui Chen, Zhangfei He, Liping Wei, Liuyan Qin, Chaosheng Li, Baoshen Li

**Affiliations:** 1 College of Resources and Environmental Sciences, China Agricultural University, Beijing, China; 2 Biotechnology Research Institute, Guangxi Academy of Agricultural Sciences, Nanning, China

**Keywords:** banana, endophytic bacteria, Fusarium wilt, microbiome, ratoon cropping, resistance

## Abstract

Fusarium wilt of banana, caused by *Fusarium oxysporum* f. sp. *cubense* (Foc), threatens global banana production. Resistant cultivars exhibit reduced disease incidence after successive ratoon cycles, but the underlying micro-ecological mechanisms remain unclear. This study represents the first longitudinal analysis of corm endophytic microbiota across ratoon cycles in banana, revealing temporal dynamics that underpin progressive disease resistance. A three-cycle field trial with three biological replicates per cultivar per cycle was conducted in a Foc-infested orchard in Guangxi, China. Corm tissues were sampled from resistant (‘Bao Dao Jiao’ and ‘Gui Jiao 9’) and susceptible ‘Williams B6’ bananas (*n* = 3 biological replicates per group) at plant crop (cycle 1) and third ratoon (cycle 3). 16S rRNA amplicons were sequenced via Illumina NovaSeq. Alpha- and beta-diversity, taxonomic composition, and predicted functions (PICRUSt2) were analyzed. Resistant cultivars maintained significantly higher Chao1 and Shannon indices than the susceptible cultivar, with divergence intensified across ratoon cycles (*p* < 0.05). Community structure was shaped primarily by cultivar rather than maternal health status. Resistant genotypes enriched *Proteobacteria* and *Actinobacteria*, alongside beneficial genera (*Halomonas*, *Nesterenkonia*, *Aliihoeflea*). Functional predictions revealed enrichment in carbohydrate metabolism, membrane transport, and xenobiotic degradation pathways in resistant cultivars. Disease incidence declined significantly from 34–39% (cycle 1) to 4–8% (cycle 3) in resistant cultivars, whereas susceptible cultivars remained at 44–59%. Resistant bananas continuously recruit beneficial endophytes during ratooning, assembling a stable microbiome that reinforces Fusarium wilt resistance. These findings provide microbial targets for breeding and biocontrol strategies.

## 1 Introduction

Banana (*Musa* spp.) is a vital cash and food crop in tropical and subtropical regions (Ploetz Randy, 2015). Ranking first among fruit crops in gross yield and international trade, it plays a critical role in food security for producing, exporting, and importing countries alike (Platonovskiy et al., 2024). Fusarium wilt, incited by Foc, is regarded as the most destructive soil-borne disease of banana, threatening production across Asia, Africa, and Latin America (Dita et al., 2018). Foc produces chlamydospores that persist in soil for >30 years, rendering chemical control largely ineffective and crop rotation economically impractical (Lin et al., 2016). Planting wilt-resistant banana varieties is an effective way to manage the disease (Dale et al., 2017).

Interestingly, long-term field monitoring indicates that resistant cultivars often display a marked decline in disease incidence from 20–40% in the first cycle to <10% after the third ratoon cycle, whereas susceptible cultivars show the opposite trend (Li et al., 2021; Zhao et al., 2024). This pattern may be linked to microbiome stabilization, wherein pathogen pressure drives the host to selectively recruit beneficial endophytes that enhance systemic resistance over successive generations (Philippot et al., 2013; Shao et al., 2025). Additionally, field observations revealed that suckers transplanted into vacant mats left by diseased plants survive better than tissue-cultured plantlets, suggesting microbial legacy effects from the previous generation that contribute to disease suppression over successive ratoons.

Endophytic microbes inhabit internal plant tissues asymptomatically. They can enhance host resistance by producing antimicrobial metabolites, competing for nutrients and niches, and priming systemic immunity (Thomas and Sekhar, 2014; Gómez-Lama Cabanás et al., 2021; Posada et al., 2024). In banana, endophyte abundance follows the gradient “root > corm > pseudostem > leaf” (Wang et al., 2014). The corm connects mother plants to suckers via vascular tissues, serving as a conduit for both pathogenic and beneficial microbes (Kaushal et al., 2020). Therefore, characterizing the endophytic bacterial communities within this compartment across cultivars and ratoon cycles may illuminate micro-ecological mechanisms underlying the observed increase in resistance.

Despite advances in high-throughput sequencing, most studies have focused on the banana rhizosphere or root endosphere. The sucker corm remains largely unexplored, particularly across multiple ratoon cycles. The corm connects mother plants to suckers via vascular tissues, serving as a conduit for both pathogenic and beneficial microbes, making it a critical but understudied compartment for understanding vertical microbiome transmission and progressive resistance. Here, we employed Illumina NovaSeq sequencing to profile the endophytic bacteria in corm tissues of resistant (‘Bao Dao Jiao’ and ‘Gui Jiao 9’) and susceptible (‘Williams B6’) bananas across cycle 1 and third-ratoon cycles. Our objective was to uncover how bacterial communities vary with host genotype and cropping duration and to identify potential microbial indicators or agents for biological control of Fusarium wilt. We hypothesized that resistant cultivars progressively enrich beneficial corm endophytes across ratoon cycles, leading to enhanced disease suppression and reduced Fusarium wilt incidence.

## 2 Materials and methods

### 2.1 Site description

The trial was conducted in Long’an County, Guangxi Zhuang Autonomous Region, China (22°0′6.80″N, 107°52′29.82″E). The site has a subtropical monsoon climate with a mean annual temperature of 22.1 °C and mean annual precipitation of 1,300 mm. The experimental field had a history of continuous banana cultivation for six ratoon cycles with susceptible cv. Williams B6 (disease incidence >45%). In November 2018, the field was fallowed, deeply ploughed and sun-dried until September 2019. Experimental plots were then established in two temporal stages: (1) Ratoon plots were planted in autumn 2019 and completed three full cycles by May 2022 (sampling time); (2) Plant-crop plots were planted in autumn 2021 for single-cycle comparison. This design ensured spatial uniformity while capturing temporal dynamics across ratoon cycles.

### 2.2 Experimental design

A randomized complete block design with three replicates was adopted. Resistant cultivars ‘Bao Dao Jiao’ (B) and ‘Gui Jiao 9’ (G), both recommended by the China Banana Industry Technology System, and susceptible ‘Williams B6’ (N) were planted. Each plot [three 25 m rows (total 140 m^2^)] contained 40 plants at 2.5 m × 1.85 m spacing. To minimize environmental heterogeneity, soil pH, organic matter, available nitrogen (N), phosphorus (P) and potassium (K) were determined by five-point sampling and did not differ significantly among plots (pH 5.8 ± 0.2, organic matter 25.4 ± 1.3 g kg^−1^, available N 45.2 ± 3.1 mg kg^−1^, available P 28.7 ± 2.5 mg kg^−1^, available K 128 ± 7 mg kg^−1^). While complete elimination of microbial carryover is impossible in field conditions, the fallow period and deep tillage in 2018–2019 aimed to reduce legacy effects, and our experimental design with randomized blocks allowed us to attribute observed microbial differences primarily to cultivar effects rather than environmental drift. All plots received identical drip fertigation; no soil fumigants or fungicides were applied. Plant-crop plots were established in autumn 2021, whereas ratoon plots originated in autumn 2019 and completed 3 cycles by sampling.

### 2.3 Sample collection and processing

**Figure 1:**
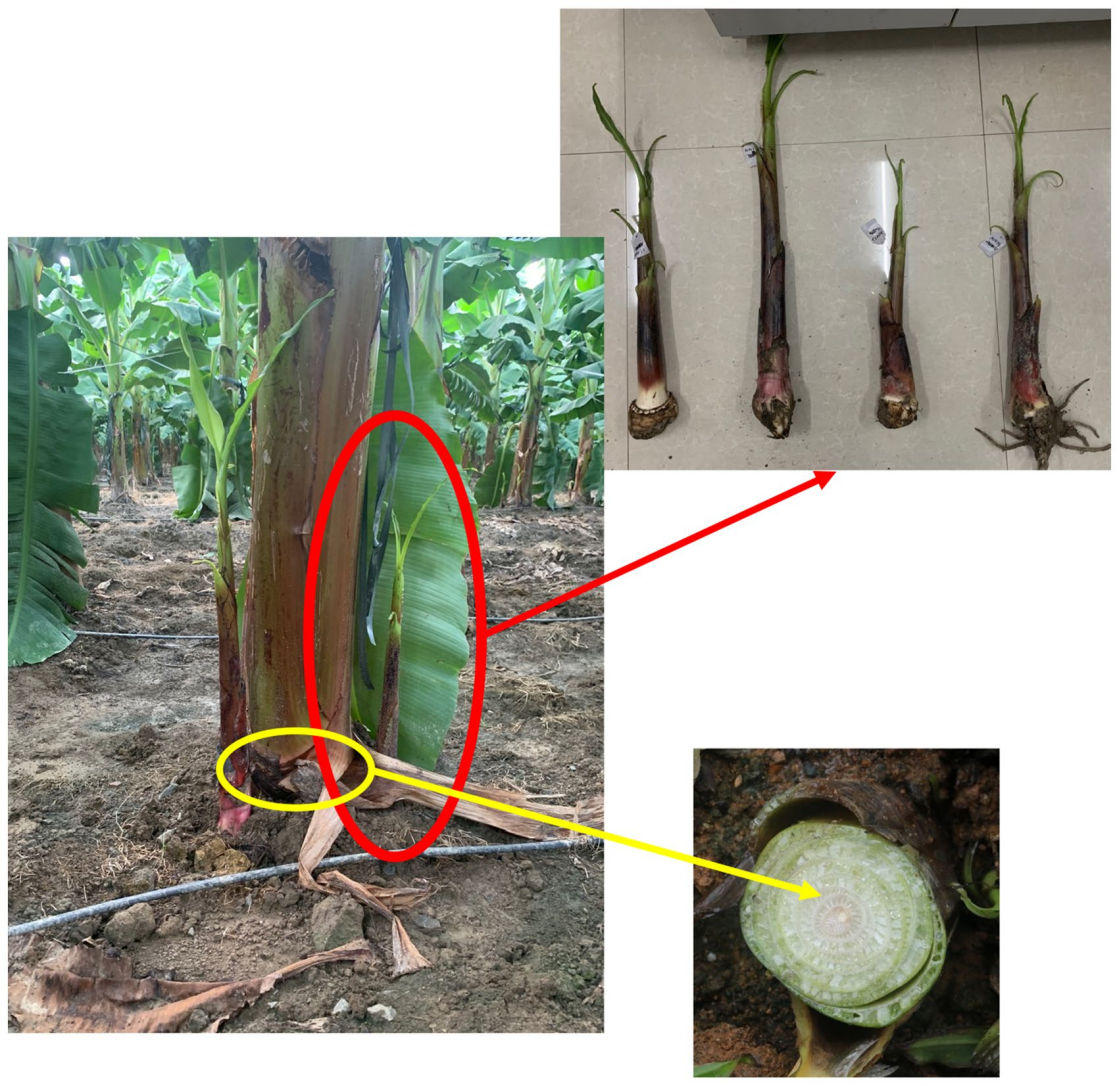
Schematic diagram of sucker corm sampling in the field.

In May 2022, sword suckers (≥15 cm diameter, Figure 1) from both healthy and diseased mats were collected per plot (three suckers pooled as one biological replicate). Corm tissue (1 cm^3^ above the apical meristem) was surface-sterilized (75% ethanol 2 min, 2% NaClO for 3 min, 75% ethanol 1 min, sterile water rinse × 3), flash-frozen in liquid nitrogen, and stored at −80 °C.

### 2.4 DNA extraction and sequencing

Genomic DNA was extracted using a DNeasy Plant Mini Kit. The V5–V7 region of the 16S rRNA gene (515F/907R) was amplified and sequenced on Illumina NovaSeq 2 × 250 bp (Personalbio, Shanghai).

### 2.5 Bioinformatics and statistics

Raw reads were demultiplexed and quality-filtered using QIIME2 v2022.2. DADA2 was used to denoise sequences and generate amplicon sequence variants (ASVs). Taxonomy was assigned using the SILVA 138 database at 99% identity. Alpha-diversity indices (Chao1, Shannon, Faith_pd) were calculated after rarefying to 50,000 reads per sample. Beta-diversity was assessed by Bray–Curtis dissimilarity and visualized by non-metric multidimensional scaling (NMDS) and unweighted pair-group method with arithmetic mean (UPGMA). Differences among groups were tested by PERMANOVA (999 permutations). LEfSe (LDA > 2.0) was used to identify biomarkers. PICRUSt2 predicted KEGG orthologs and pathways. Statistical analyses were performed in SPSS 20.0 using one-way ANOVA followed by Duncan’s multiple-range test (*p* < 0.05).

### 2.6 Disease assessment

From the onset of symptoms, plants were inspected weekly. Disease incidence (%) = (symptomatic plants / total plants) × 100 was recorded at harvest (Table 1).

**Table 1:** Experimental treatments and blight incidence in different treatments.

Variety	Average incidence rate	Mother plant health status	Planting years	Treatment name
Williams B6	59.2% ± 7.6%	Healthy	1	NN1
Williams B6		Diseased	1	NX1
Gui jiao 9	34.5% ± 9.5%	Healthy	1	GN1
Gui jiao 9		Diseased	1	GX1
Bao Dao Jiao	39.4% ± 13.2%	Healthy	1	BN1
Bao Dao Jiao		Diseased	1	BX1
Williams B6	44.3% ± 8.0%	Healthy	3	NNS
Williams B6		Diseased	3	NXS
Gui jiao 9	8.3% ± 7.8%	Healthy	3	GNS
Gui jiao 9		Diseased	3	GXS
Bao Dao Jiao	4.3% ± 1.7%	Healthy	3	BNS
Bao Dao Jiao		Diseased	3	BXS

## 3 Results

### 3.1 Sequencing output and data quality

**Figure 2:**
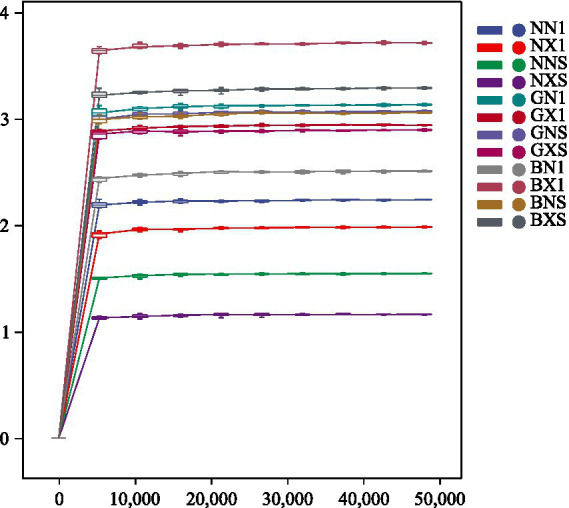
Rarefaction curves of corm endophytes.

**Figure 3:**
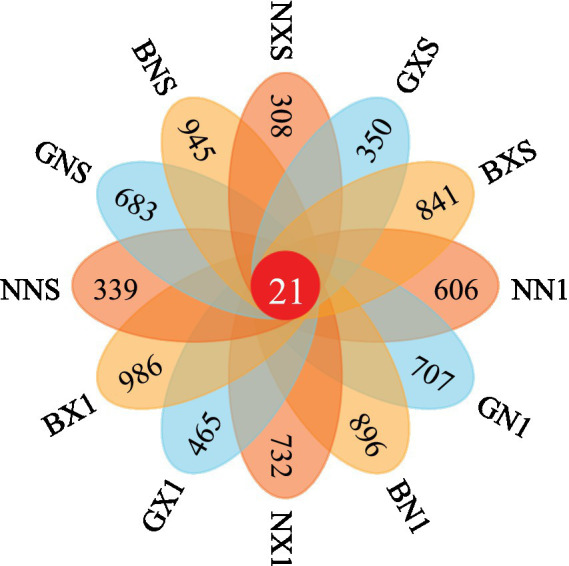
Venn diagram of unique ASVs in corm endophytes across cycles 1 and 3.

After DADA2 quality filtering, a total of 2,847,252 high-quality sequences were retained, with a mean of 61,374 ± 8,926 sequences per sample. Rarefaction curves reached a plateau at 50,000 sequences (Figure 2), indicating sufficient sequencing depth. Figure 3 shows that in cycle 1 corm tissues, the number of unique ASVs followed the order: diseased Bao Dao Jiao (BX1) > healthy Bao Dao Jiao (BN1) > diseased Williams B6 (NX1) > healthy Gui Jiao 9 (GN1) > healthy Williams B6 (NN1) > diseased Gui Jiao 9 (GX1). In cycle 3 corm tissues, the order was BNS > BXS > GNS > GXS > NNS > NXS. This Venn diagram demonstrates that resistant cultivars accumulated more unique ASVs between cycles 1 and 3 compared to susceptible lines, suggesting greater microbial innovation and colonization capacity in resistant genotypes.

### 3.2 Alpha diversity

**Figure 4:**
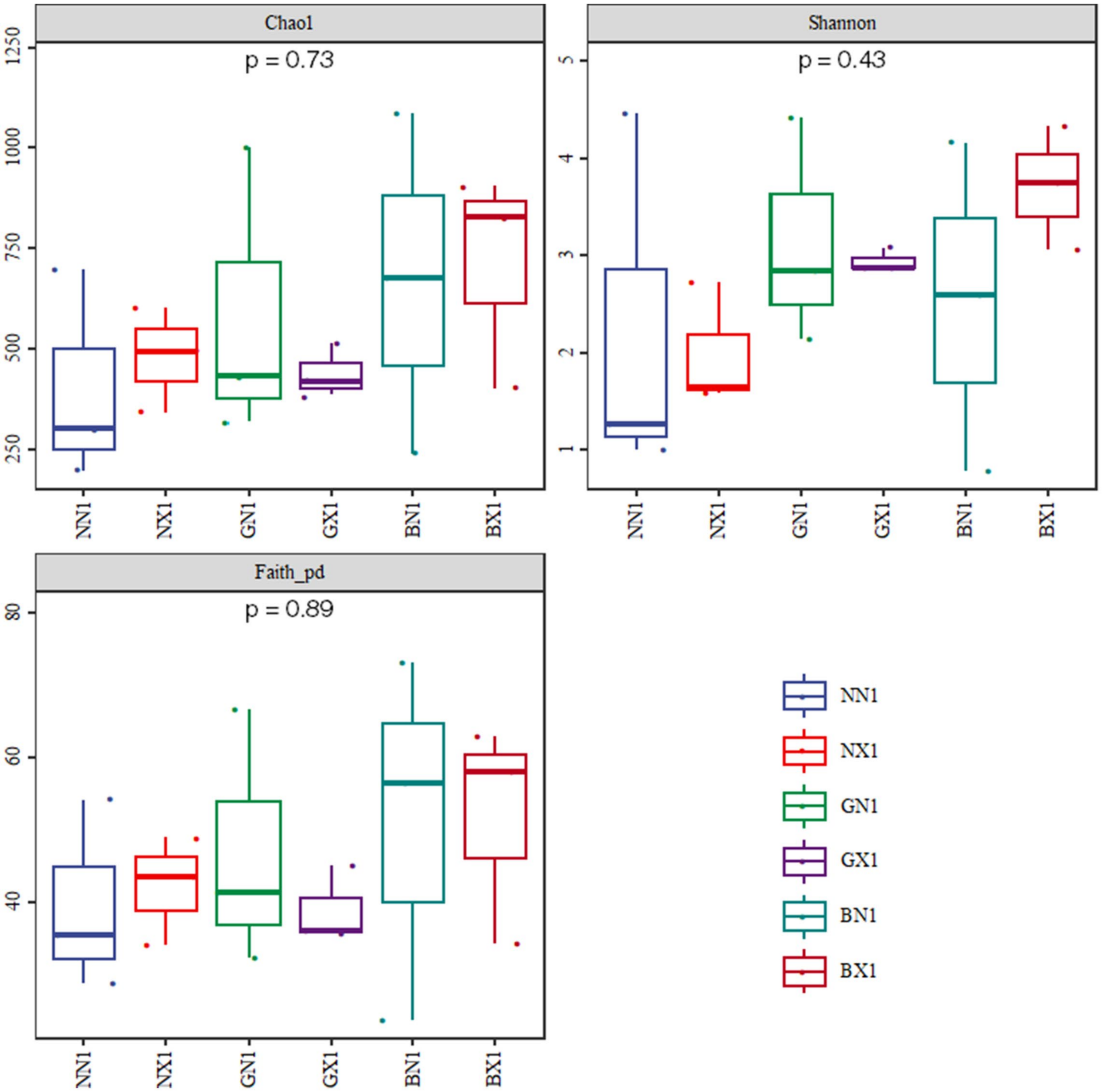
Alpha diversity of endophytic bacteria in the cycle 1 of corm tissues.

**Figure 5:**
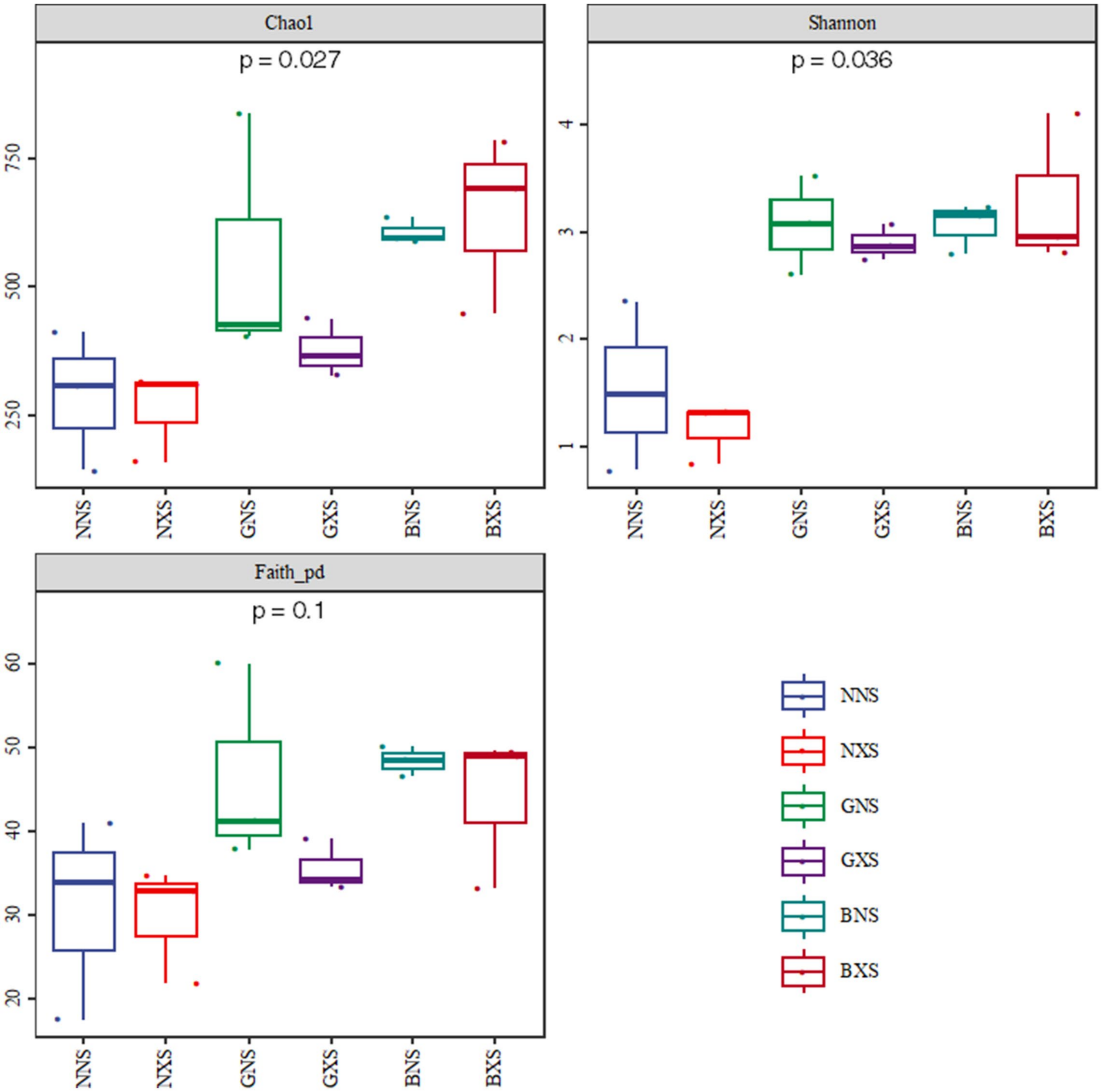
Alpha diversity of endophytic bacteria in cycle 3 of different varieties.

In cycle 1, the median Chao1, Shannon, and Faith_pd indices of resistant varieties were higher than those of the susceptible variety, though differences were modest; whereas in cycle 3, the Chao1 index and Shannon index differed significantly between the susceptible and disease-resistant varieties (*p* < 0.05). Disease-resistant varieties continued to enrich new colonies during the continuous cropping process, while susceptible varieties showed a declining trend, and resistant cultivars exhibited greater taxonomic richness and functional potential, enabling exploitation of diverse metabolic resources (Figures 4, 5).

### 3.3 Beta diversity

Based on Bray-Curtis distances, NMDS ordination for Cycle 1 samples exhibited a stress value of 0.0247, with PERMANOVA revealing significant independent effects of both host genotype (*R*^2^ = 0.321, *p* = 0.001) and disease status (*R*^2^ = 0.148, *p* = 0.002) on root-associated microbiome structure. For third-ratoon samples, NMDS fit improved substantially (stress = 0.0074), and PERMANOVA indicated that genotype (susceptible vs. resistant) overwhelmingly drove microbial community variation (*R*^2^ = 0.789, *p* < 0.001), while disease status exerted a smaller but significant effect (*R*^2^ = 0.112, *p* = 0.001) without genotype × status interaction, demonstrating that disease-resistant varieties maintain a stable microbiome irrespective of disease infestation. Corm tissues of healthy and susceptible mother plants of the same variety were closer on the NMDS plot, but the distance was significantly larger across varieties, indicating that varietal differences > differences in the health status of mother plants. The clustering tree showed that all banana were clustered into one unit and susceptible samples into another, further confirming cultivar-specific community convergence over time.

### 3.4 Taxonomic composition

#### 3.4.1 Phylum level

**Figure 6:**
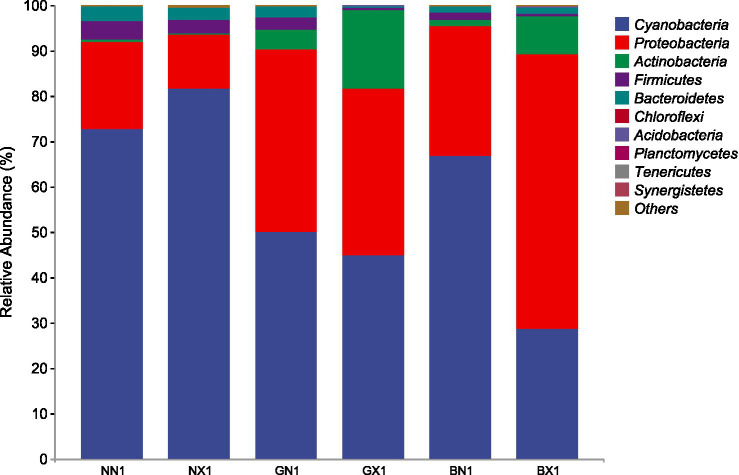
Stacked bar chart of relative abundance of endophytes at the phylum level in different banana varieties of the cycle 1.

**Figure 7:**
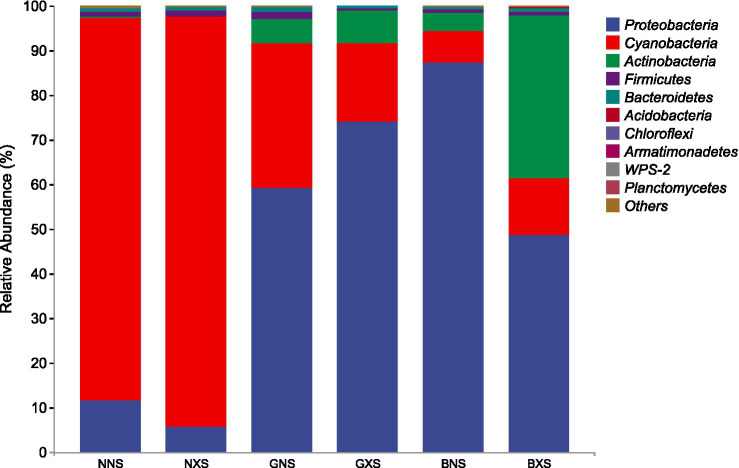
Stacked bar chart of relative abundance of endophytes at the phylum level in different banana varieties of cycle 3.

Figures 6, 7 show the differences in the relative abundance of endophytes at the phylum level for different banana cultivar cycle 1 s and cycle 3 corm tissues, respectively. The results showed that Proteobacteria and Cyanobacteria were the dominant phyla in the cycle 1 and cycle 3, but the abundance of Cyanobacteria was relatively higher in the susceptible varieties, whereas the abundance of Proteobacteria was significantly higher in the disease-resistant varieties, and the difference was more pronounced in the cycle 3. Actinobacteria was the third dominant phylum in relative abundance, and its relative abundance was higher in the disease-resistant varieties and significantly higher in the susceptible group than in the healthy group.

The dominance of Proteobacteria in resistant varieties is particularly noteworthy. This phylum is known for its diverse metabolic capabilities, including production of antimicrobial compounds, siderophores, and plant growth hormones that can suppress pathogens. The significantly higher abundance of Proteobacteria in resistant cultivars (48.61–87.33% in cycle 3 vs. 5.76–11.54% in susceptible cultivars, Figure 7) suggests they may actively contribute to disease suppression through competitive exclusion and induced systemic resistance.

#### 3.4.2 Genus level

**Figure 8:**
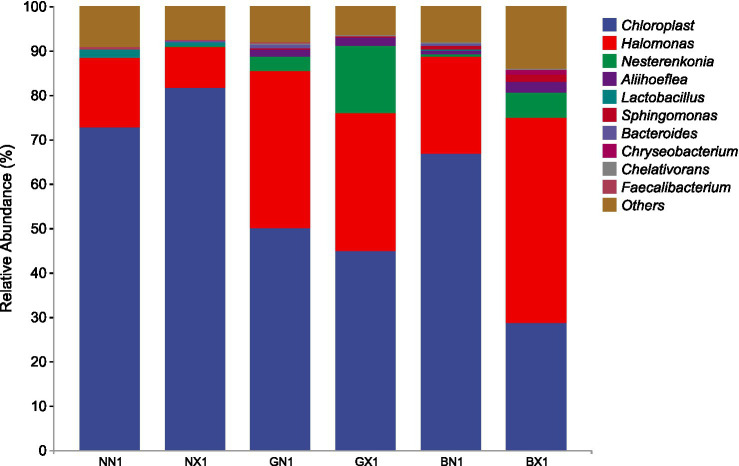
Stacked bar chart of relative abundance of endophytes at the genus level in the cycle 1 of different banana varieties.

**Figure 9:**
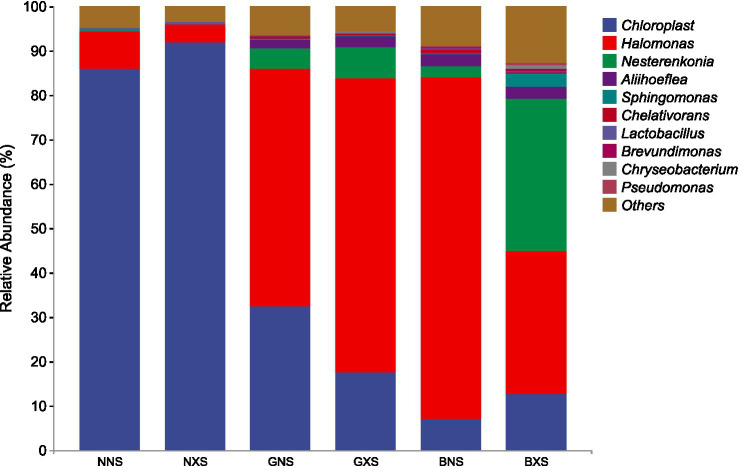
Stacked bar chart of relative abundance of endophytes at the genus level in cycle 3 of different banana varieties.

The results showed that *Chloroplast* and *Halomonas* were the dominant genera in both cycle 1 and cycle 3, with Chloroplast having relatively high abundance in susceptible varieties and Halomonas having significantly higher abundance in disease-resistant varieties, and the difference was even more pronounced in cycle 3. Nesterenkonia and Aliihoeflea were the third and fourth dominant genera in relative abundance, and their relative abundance in disease-resistant varieties was higher than that in cycle 3, which were higher in the disease-resistant varieties, but the relative abundance of Nesterenkonia was significantly higher in the susceptible group than in the healthy group (Figures 8, 9). The enrichment of these genera in resistant cultivars suggests their potential as biocontrol agents, though strain-specific functional validation is required to confirm their protective capacity.

### 3.5 Analysis of significantly different bacterial populations

**Figure 10:**
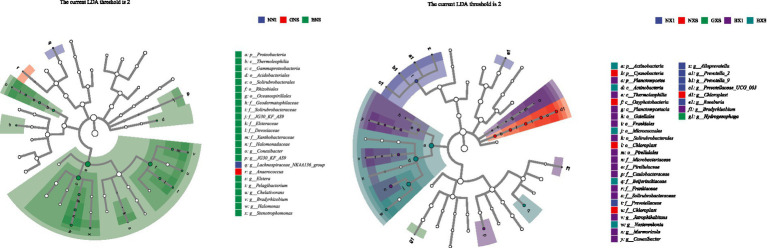
Tree-like cladogram of LEfSe analysis of endophytes in different varieties of the cycle 1 (left) and cycle 3 (right). The circles radiating from the inside outward represent taxonomic levels from phylum to species. Each small circle at each taxonomic level represents a taxonomic unit at that level, with the diameter of the small circle proportional to the relative abundance of the species.

**Figure 11:**
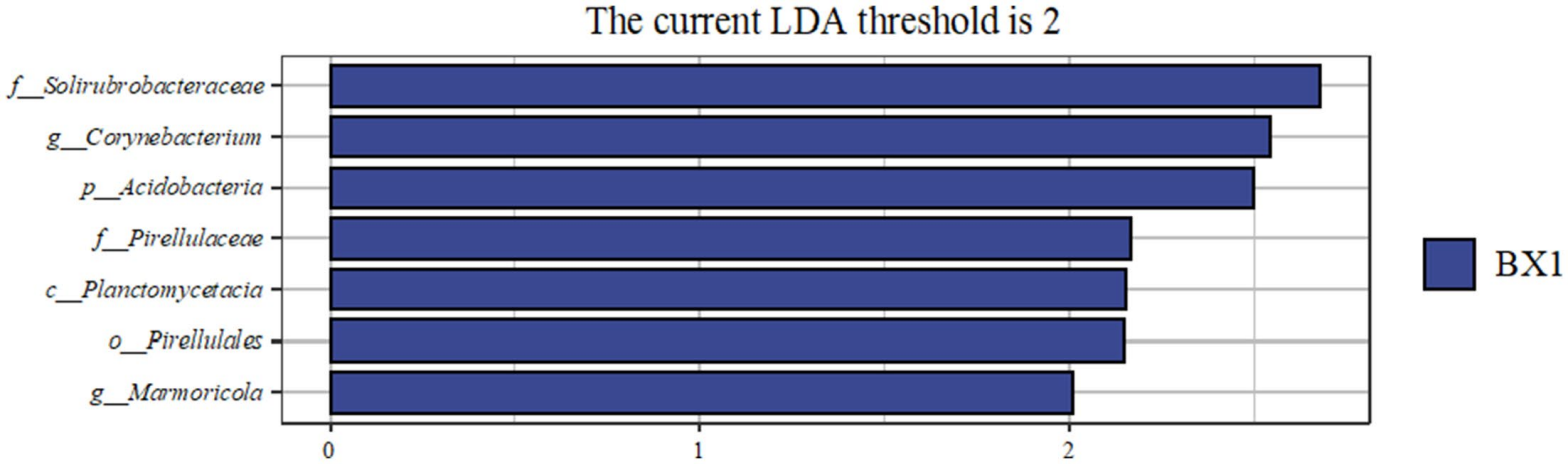
Histogram of LDA values from LEfSe analysis of endophytes in the cycle 1 of different varieties. The length of the histogram bars indicates the degree of influence of species with significant differences.

**Figure 12:**
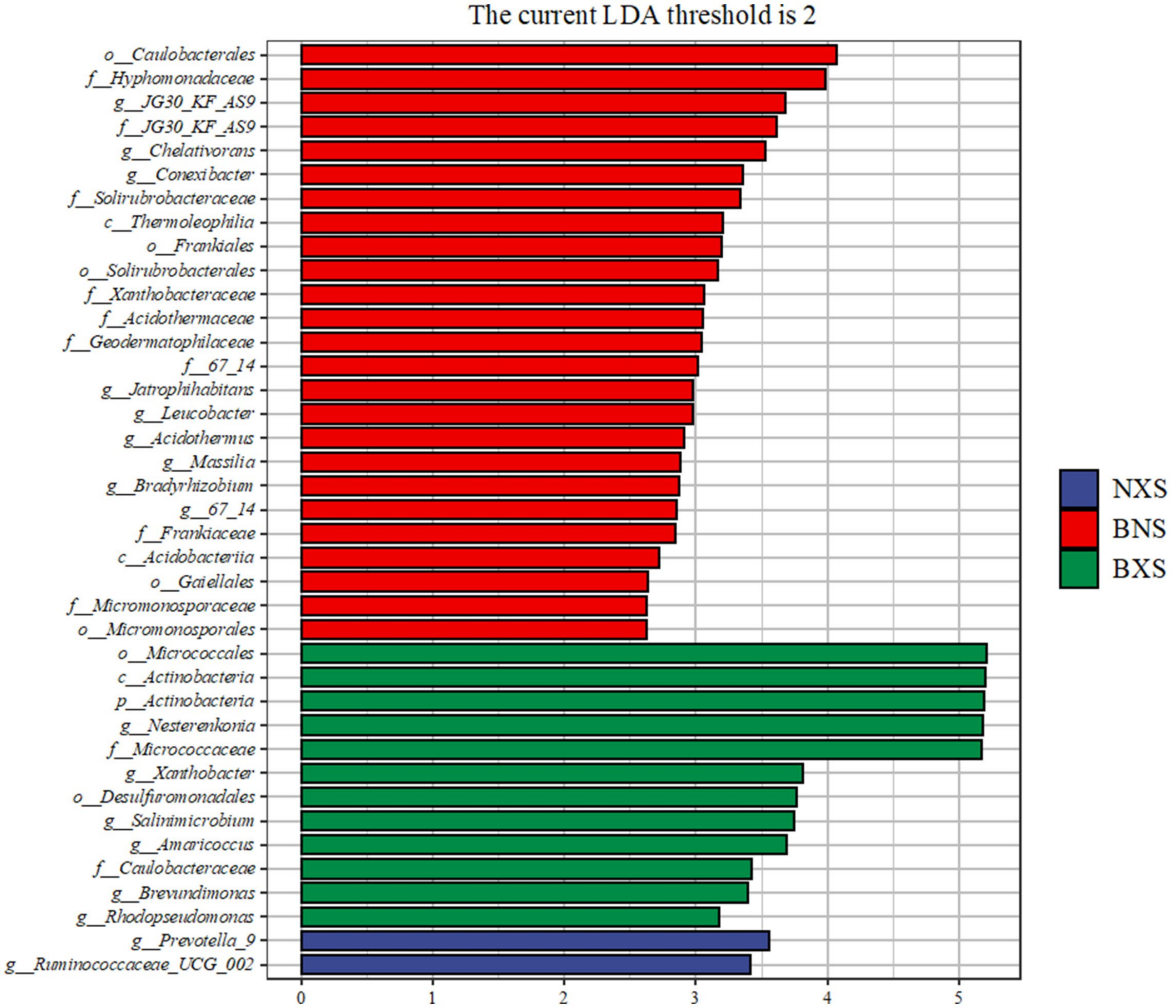
Histogram of LDA values from LEfSe analysis of endophytes in cycle 3 of different varieties.

Under the condition of LDA of 2, the results of LEfSe analysis showed that the bacterial community structure of the BX1 group in the cycle 1 samples was significantly different from the other groups, whereas the bacterial community structure of the NXS, BNS, and BXS groups in the host plantain samples was more complex, involving significant enrichment of several phyla and genera. The BX1 group was enriched with Corynebacterium in the cycle 1; the BNS group was enriched with 12 genera such as Chelativorans, Conexibacter, Jatrophihabitans, and Bradyrhizobium in the host plantain, and the NXS group was enriched with Prevotella, Anaerococcus, and other Anaerococcus. These different groups may be directly related to the host disease resistance (Figures 10–12).

### 3.6 Functional prediction based on PICRUSt2

**Figure 13:**
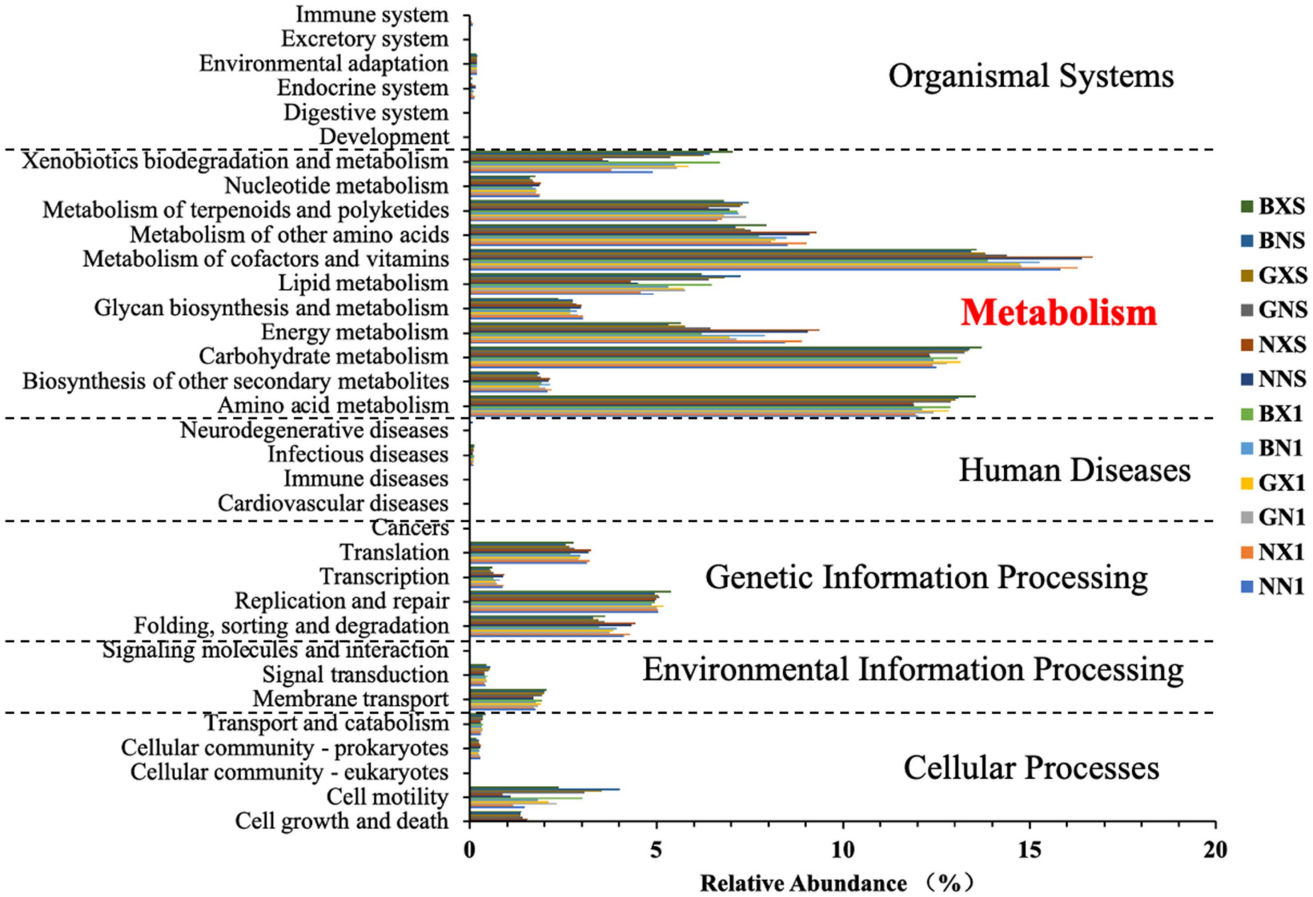
Functional prediction of endophytic bacteria based on PICRUSt2 (level 2 KEGG pathways).

As shown in Figure 13, endophytic bacterial functions were primarily focused on metabolism, cellular processes, environmental information processing, and genetic information processing. The metabolism category had the highest relative abundance, particularly amino acid and carbohydrate metabolism, which may be related to the growth and energy requirements of the samples. Genetic information processing was the next highest, but the higher abundance of folding, sorting, degradation, translation, and replication and repair functions suggests that the samples also play an important role in maintaining the stability and function of genetic information.

Functions with an average relative abundance greater than 1% were selected for further analysis in the secondary functional classification. Notable differences were observed in specific metabolic pathways. Carbohydrate metabolism was significantly enriched in resistant cultivars (13.39% in BNS vs. 12.33% in NNS, *p* < 0.05, Table 2). Membrane transport (ABC transporters) showed 2.00% abundance in resistant cultivars compared to 1.70% in susceptible cultivars in cycle 3 (*p* < 0.05, Table 3). Additionally, xenobiotic biodegradation pathways were significantly higher in resistant varieties (6.43% in BNS vs. 3.72% in NNS, *p* < 0.05).

**Table 2:** Predicted functional capacity in different functions under the metabolism classification in endophyte samples of different treatments (%).

Level1	Metabolism										
Level2	Amino acid metabolism	Biosynthesis of other secondary metabolites	Carbohydrate metabolism	Energy metabolism	Glycan biosynthesis and metabolism	Lipid metabolism	Metabolism of cofactors and vitamins	Metabolism of other amino acids	Metabolism of terpenoids and polyketides	Nucleotide metabolism	Xenobiotics biodegradation and metabolism
NN1	12.01 ± 0.27b	2.08 ± 0.10ab	12.49 ± 0.40bc	8.44 ± 1.39ab	3.02 ± 0.09a	4.91 ± 0.99b	15.83 ± 1.21a	8.51 ± 0.60ab	6.62 ± 0.72a	1.86 ± 0.02a	4.90 ± 0.28b
NX1	11.94 ± 0.15b	2.18 ± 0.08a	12.40 ± 0.15c	8.90 ± 0.47a	3.03 ± 0.07a	4.58 ± 0.29b	16.29 ± 0.37a	9.03 ± 0.27a	6.76 ± 0.43a	1.88 ± 0.02a	3.78 ± 0.12c
GN1	12.42 ± 0.34ab	2.02 ± 0.01ab	12.79 ± 0.35abc	7.15 ± 1.07abc	2.89 ± 0.09b	5.76 ± 0.82ab	14.78 ± 0.86ab	8.07 ± 0.56b	7.41 ± 0.37a	1.76 ± 0.04b	5.54 ± 0.35b
GX1	12.83 ± 0.27a	1.85 ± 0.06c	13.14 ± 0.27a	6.96 ± 0.34bc	2.68 ± 0.06c	5.72 ± 0.08ab	14.74 ± 0.24ab	8.2 ± 0.12ab	6.81 ± 0.49a	1.78 ± 0.01b	5.85 ± 0.21ab
BN1	12.12 ± 0.44b	2.14 ± 0.11a	12.42 ± 0.36c	7.90 ± 1.35abc	2.86 ± 0.06b	5.32 ± 0.97ab	15.27 ± 1.23ab	8.49 ± 0.66ab	7.20 ± 0.54a	1.76 ± 0.09b	5.49 ± 0.92b
BX1	12.88 ± 0.22a	1.92 ± 0.12bc	13.08 ± 0.35ab	6.21 ± 0.52c	2.69 ± 0.08c	6.49 ± 0.25a	13.88 ± 0.41b	7.75 ± 0.30b	7.16 ± 0.29a	1.67 ± 0.03b	6.7 ± 0.61a
NNS	11.90 ± 0.07b	2.11 ± 0.08ab	12.33 ± 0.07b	9.05 ± 0.65a	2.97 ± 0.04a	4.50 ± 0.34c	16.40 ± 0.60a	9.10 ± 0.40a	6.95 ± 0.65ab	1.86 ± 0.05a	3.72 ± 1.13c
NXS	11.88 ± 0.05b	2.15 ± 0.03a	12.31 ± 0.04b	9.37 ± 0.25a	2.99 ± 0.02a	4.31 ± 0.12c	16.69 ± 0.21a	9.30 ± 0.16a	6.40 ± 0.27b	1.89 ± 0.02a	3.55 ± 0.62c
GNS	12.89 ± 0.22a	1.9 ± 0.14abc	13.26 ± 0.21a	6.44 ± 0.81b	2.84 ± 0.03a	6.40 ± 0.61b	14.39 ± 0.61b	7.53 ± 0.61bc	7.24 ± 0.42ab	1.72 ± 0.05b	5.36 ± 0.67b
GXS	13.01 ± 0.33a	1.8 ± 0.05c	13.35 ± 0.31a	5.76 ± 0.63b	2.75 ± 0.06a	6.83 ± 0.45ab	13.81 ± 0.53b	7.36 ± 0.26bc	7.31 ± 0.37ab	1.67 ± 0.02bc	6.27 ± 0.32ab
BNS	13.09 ± 0.15a	1.85 ± 0.15abc	13.39 ± 0.14a	5.31 ± 0.32b	2.76 ± 0.03a	7.26 ± 0.26a	13.43 ± 0.22b	7.11 ± 0.16c	7.48 ± 0.35a	1.61 ± 0.02c	6.43 ± 0.14ab
BXS	13.55 ± 0.85a	1.82 ± 0.32bc	13.72 ± 0.73a	5.66 ± 0.73b	2.36 ± 0.38b	6.22 ± 0.66b	13.58 ± 0.82b	7.94 ± 0.35b	6.81 ± 0.65ab	1.75 ± 0.12b	7.05 ± 1.04a

Values are mean ± SD; different letters indicate significant differences (*p* < 0.05, Duncan’s test).

**Table 3:** Predicted functional capacity in different functions in endophyte samples of different treatments (%).

Level1	Cellular processes		Environmental information processing	Genetic information processing		
Level2	Cell growth and death	Cell motility	Membrane transport	Folding, sorting and degradation	Replication and repair	Translation
NN1	1.52 ± 0.04a	1.46 ± 0.91b	1.75 ± 0.11bc	4.12 ± 0.47a	5.04 ± 0.17ab	3.14 ± 0.02a
NX1	1.54 ± 0.02a	1.15 ± 0.33b	1.71 ± 0.02c	4.28 ± 0.17a	5.02 ± 0.03ab	3.20 ± 0.06a
GN1	1.44 ± 0.04b	2.32 ± 0.66ab	1.82 ± 0.08abc	3.75 ± 0.31b	5.01 ± 0.09ab	2.91 ± 0.13bc
GX1	1.42 ± 0.02b	2.11 ± 0.10ab	1.9 ± 0.05ab	3.84 ± 0.03b	5.18 ± 0.12a	2.93 ± 0.01b
BN1	1.48 ± 0.05b	1.82 ± 0.99ab	1.76 ± 0.11bc	3.92 ± 0.46b	4.87 ± 0.02b	2.95 ± 0.23b
BX1	1.41 ± 0.05b	3.01 ± 0.33a	1.93 ± 0.06a	3.46 ± 0.07b	4.95 ± 0.16b	2.68 ± 0.07c
NNS	1.52 ± 0.02a	1.08 ± 0.37c	1.70 ± 0.02b	4.33 ± 0.23a	4.96 ± 0.05b	3.18 ± 0.12a
NXS	1.56 ± 0.02a	0.86 ± 0.12c	1.69 ± 0.01b	4.44 ± 0.09a	4.99 ± 0.02ab	3.25 ± 0.04a
GNS	1.41 ± 0.04b	3.06 ± 0.65ab	1.93 ± 0.06a	3.6 ± 0.22b	5.08 ± 0.03ab	2.8 ± 0.13b
GXS	1.36 ± 0.02bc	3.54 ± 0.42a	1.98 ± 0.07a	3.44 ± 0.09b	5.04 ± 0.08ab	2.67 ± 0.08bc
BNS	1.35 ± 0.02c	4.01 ± 0.31a	2.00 ± 0.03a	3.3 ± 0.04b	4.95 ± 0.02b	2.56 ± 0.05c
BXS	1.37 ± 0.04bc	2.38 ± 0.94b	2.05 ± 0.15a	3.63 ± 0.33b	5.38 ± 0.49a	2.77 ± 0.21bc

Values are mean ± SD; different letters indicate significant differences (*p* < 0.05, Duncan’s test).

In metabolism, the trend of gene sequence expression differences between healthy and susceptible plants of susceptible varieties in the cycle 1 differed significantly from that among resistant varieties. For example, in carbohydrate metabolism, biodegradation, and metabolism of xenobiotics, GX1 was significantly higher than GN1, and BX1 was also significantly higher than BN1, but NX1 was significantly lower than NN1. In cycle 3, differences between healthy and susceptible strains of susceptible varieties were relatively small, but there were significant differences in the expression of several metabolic gene sequences between susceptible and resistant varieties.

In cellular processes, the abundance of susceptible varieties was significantly higher than that of resistant varieties in cell growth and death, while the opposite was true for cell motility. There were no significant differences between healthy and susceptible plants of any susceptible variety.

In environmental information processing, the abundance of membrane transport was lower in all susceptible varieties than in resistant varieties, with significant differences in cycle 3. Meanwhile, in cycle 1, susceptible plants of resistant varieties were all significantly higher than healthy plants in membrane transport abundance.

In genetic information processing, the abundance of folding, sorting and degradation, and translation in susceptible varieties were all significantly higher than in resistant varieties, with no significant differences between healthy and susceptible plants of susceptible varieties.

## 4 Discussion

### 4.1 Diversity and stability

The progressive increase in alpha-diversity in resistant cultivars supports the ‘cry-for-help’ hypothesis (Beltrán-García et al., 2021), whereby sustained pathogen pressure may enable plants to selectively enrich beneficial microbes, fostering a more robust defensive microbiome across ratoon cycles. UPGMA clustering indicated that resistant ratoons developed a more stable and convergent community structure, whereas susceptible plants exhibited increasingly divergent or declining communities—a phenomenon similarly observed in other perennial systems (Nakkeeran et al., 2021). Intriguingly, resistant varieties maintained this capacity for beneficial enrichment even when sourced from diseased mother plants, pointing to genotype-specific microbial filtering mechanisms. While our findings are compatible with cry-for-help dynamics, we acknowledge that direct evidence of active plant signaling remains elusive (Mon et al., 2021; Lu et al., 2025). Future work integrating metabolomic analysis of corm exudates with transcriptomic profiling of host defense genes will be essential to validate these mechanisms and establish causality. This functional redundancy ensures that if one protective taxon declines, others can compensate, maintaining consistent pathogen suppression. Additionally, many enriched taxa produce antifungal metabolites or compete for niche space, directly inhibiting Foc colonization.

### 4.2 Keystone taxa and their putative functions

The dominance of *Proteobacteria* and *Actinobacteria* in resistant varieties is particularly noteworthy. *Proteobacteria,* the dominant phylum across all compartments, is known for its diverse metabolic capabilities and ecological functions, including nutrient cycling, plant growth promotion, and disease suppression. For instance, Alghamdi et al. (2024) reported that *Proteobacteria* can mitigate salt stress and enhancing plant tolerance. Ebrahimiz-Zarandi et al. (2022) highlighted the potential of Actinobacteria to produce biocontrol agents that can suppress plant pathogens. Our data demonstrate this enrichment quantitatively: *Proteobacteria* abundance increased from 5.76–11.54% in cycle 1 to 48.61–87.33% in cycle 3 in resistant cultivars (Figure 7), while *Halomonas* relative abundance was 6.2–8.9-fold higher in healthy resistant vs. susceptible cultivars in healthy cycle 3 (Figure 9). The observed enrichment of *Halomonas* in resistant varieties further supports this hypothesis, as this genus has been implicated in biocontrol due to its salt tolerance and antimicrobial properties. *Halomonas* species have been shown to produce exopolysaccharides that can protect plants from pathogen invasion and environmental stresses (Mukherjee et al., 2019; Meinzner et al., 2023; Liu et al., 2025). However, the biocontrol effect of *Halomonas* and related strains and their interactions with the host need to be verified in the future through the sterile seedling splice-back test, as genus-level identification may mask strain-specific functional variation. Collectively, these traits enable direct pathogen inhibition and priming of host systemic immunity, reinforcing corm-specific defense.

Conversely, the higher abundance of Cyanobacteria in susceptible varieties raises concerns. While Cyanobacteria can fix nitrogen, their dominance in susceptible varieties may indicate an imbalance in the endophytic community that favors less beneficial or even harmful microorganisms. This imbalance could compromise the plant’s ability to resist pathogen invasion, as suggested by Santoyo et al. (2016) who noted that an overabundance of certain microbial groups can lead to dysbiosis, making plants more vulnerable to diseases.

**Figure 14:**
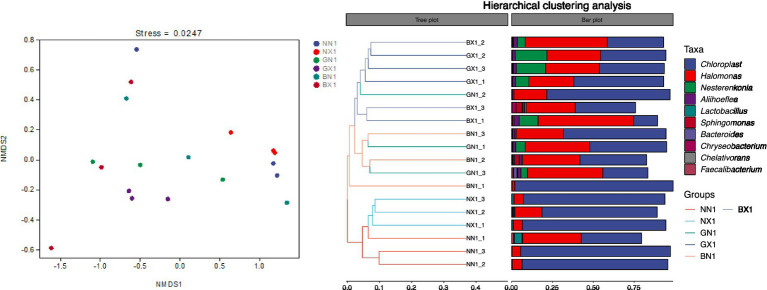
NMDS analysis (left) and hierarchical clustering analysis (right) of beta diversity of endophytes in the cycle 1 of corm tissues of different species.

**Figure 15:**
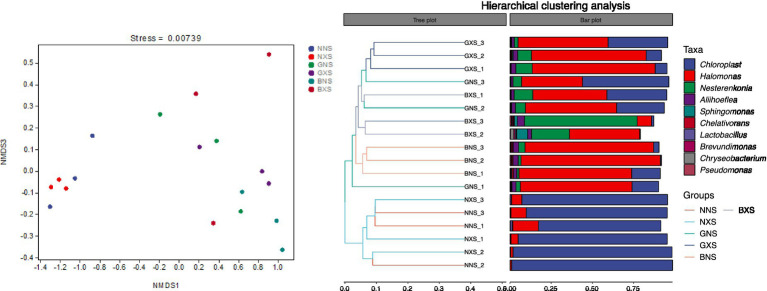
NMDS analysis (left) and hierarchical cluster analysis (right) of beta diversity of endophytes of different varieties of third-ratoon suckers.

The corm represents a unique and critical compartment for microbiome-mediated resistance. Unlike the rhizosphere, which is subject to constant environmental fluctuations, the corm serves as a vegetative propagation organ that transmits endophytes vertically from mother plants to suckers via vascular connections. This vertical transmission creates a microbial legacy effect, allowing beneficial bacteria to be inherited across generations and establish stable, protective consortia before the sucker emerges into the pathogen-laden soil environment. Our findings that cultivar effects outweigh maternal health status (Figures 14, 15) support this mechanism, suggesting genotype-specific filters shape a core microbiome in this crucial transmission organ.

### 4.3 Functional pathways

Functional predictions based on PICRUSt2 analysis reveal that resistant varieties exhibit unique advantages in metabolic processes such as amino acid and carbohydrate metabolism. These metabolic functions are crucial for plant growth and energy production, suggesting that endophytes in resistant varieties may enhance plant vigor and stress tolerance. By comparing the metabolic functions of endophytes from different resistant sugarcane varieties, Chen et al. (2023) found that endophytes from resistant varieties showed significant enrichment in metabolic pathways, which were not only sufficient to provide plants with the necessary energy and material base, but also enhanced plant growth and stress resistance. Additionally, Aqueel et al. (2023) found that advantages of resistant varieties in metabolic processes such as amino acid metabolism and carbohydrate metabolism may enhance plant vigor and tolerance. These enriched metabolic pathways likely fuel production of defense-related compounds and energy-intensive immune responses, thereby enhancing basal resistance.

It is critical to emphasize that PICRUSt2 predictions are based on 16S rRNA gene sequences and represent putative functional capacity, not confirmed metabolic activity. These inferences require validation through direct metabolomic profiling, transcriptomics, or biochemical assays to confirm that predicted pathways are actively expressed. Without such experimental confirmation, these functional interpretations remain hypothetical.

## 5 Conclusion

This study provides the first comprehensive evidence that resistant banana cultivars progressively assemble a stable, highly diverse corm endophytic microbiome across ratoon cycles, leading to significant disease suppression. The divergence in community structure driven by cultivar genotype, enrichment of *Proteobacteria* and beneficial genera (*Halomonas*, *Nesterenkonia*), and enhanced metabolic pathways collectively form a ‘three-high’ micro-ecosystem characterized by high diversity, functional redundancy, and stability. While this field study was conducted at a single location—where prior high Fusarium incidence ensured robust pathogen pressure but may also have introduced residual microbial legacies—multi-site validation is needed to confirm robustness across different soil types and Foc inoculum pressures. Future research may integrate multi-omics approaches to validate these findings, including metabolomic analysis of corm exudates, isotope-tracing experiments to confirm vertical transmission, and controlled Foc challenge experiments with gnotobiotic plantlets inoculated with synthetic communities of identified keystone taxa. Long-term field trials tracking microbiome stability beyond three ratoon cycles will also be essential for developing durable biocontrol strategies, providing theoretical foundation for endophyte-based green prevention technologies against Fusarium wilt.

## Data Availability

The sequencing data of the 16S rRNA gene in this study are available in the Sequence Read Archive (SRA) under project number PRJNA1304191.

## References

[ref1] Alghamdi A. K. Parween S. Hirt H. Saad M. M. (2024). Unveiling the bacterial diversity and potential of the *Avicennia marina* ecosystem for enhancing plant resilience to saline conditions. Environ. Microbiome 19:101. doi: 10.1186/s40793-024-00642-w, 39633419 PMC11619459

[ref2] Aqueel R. Badar A. Roy N. Mushtaq Q. Ali A. F. Bashir A. . (2023). Cotton microbiome profiling and cotton leaf curl disease suppression through microbial consortia associated with *Gossypium arboreum*. NPJ Biofilms Microbiomes 9:100. doi: 10.1038/s41522-023-00470-9 38097579 PMC10721634

[ref3] Beltrán-García M. J. Martínez-Rodríguez A. Olmos-Arriaga I. Valdez-Salas B. Chavez-Castrillon Y. Y. Di Mascio P. . (2021). Probiotic endophytes for more sustainable banana production. Microorganisms 9:1927. doi: 10.3390/microorganisms9091805 34576701 PMC8469954

[ref4] Chen S. Chen Z. Lin X. Zhou X. Yang S. Tan H. (2023). Why different sugarcane cultivars show different resistant abilities to smut? Comparisons of endophytic microbial compositions and metabolic functions in stems of sugarcane cultivars with different abilities to resist smut. BMC Plant Biol. 23:427. doi: 10.1186/s12870-023-04446-x, 37710150 PMC10500793

[ref5] Dale J. James A. Paul J.-Y. Khanna H. Smith M. Peraza-Echeverria S. . (2017). Transgenic Cavendish bananas with resistance to Fusarium wilt tropical race 4. Nat. Commun. 8:1496. doi: 10.1038/s41467-017-01670-6, 29133817 PMC5684404

[ref6] Dita M. Barquero M. Heck D. Mizubuti E. S. G. Staver C. P. (2018). Fusarium wilt of banana: current knowledge on epidemiology and research needs toward sustainable disease management. Front. Plant Sci. 9:1468. doi: 10.3389/fpls.2018.01468 30405651 PMC6202804

[ref7] Ebrahimiz-Zarandi M. Saberi Riseh R. Tarkka M. T. (2022). Actinobacteria as effective biocontrol agents against plant pathogens: an overview of their role in eliciting plant defense. Microorganisms 10:1739. doi: 10.3390/microorganisms10091739 36144341 PMC9500821

[ref8] Gómez-Lama Cabanás C. Fernández-González A. J. Cardoni M. Valverde-Corredor A. López-Cepero J. Fernández-López M. . (2021). The banana root endophytome: differences between mother plants and suckers and evaluation of selected bacteria to control *Fusarium oxysporum* f. sp. *cubense*. J. Fungi 7:224. doi: 10.3390/jof7030194 PMC8002102 33803181

[ref9] Kaushal M. Mahuku G. Swennen R. (2020). Metagenomic insights of the root-colonizing microbiome associated with symptomatic and non-symptomatic bananas in Fusarium wilt-infected fields. Plants 9:251. doi: 10.3390/plants9020263 32085593 PMC7076721

[ref10] Li C. Tian D. Wei S. Li B. Li J. Wei D. . (2021). Planting performance of five different banana disease-resistant varieties (lines) in severely affected areas of Guangxi with Fusarium wilt disease. South China Fruits 50, 76–79. doi: 10.13938/j.issn.1007-1431.20210135

[ref11] Lin Y.-H. Lin Y.-J. Chang T.-D. Hong L.-L. Chen T.-Y. Chang P.-F. L. . (2016). Development of a TaqMan probe-based insulated isothermal PCR assay for detection of *Fusarium oxysporum* f. sp. *cubense* race 4. PLoS One 11:e0159681. doi: 10.1371/journal.pone.0159681 27448242 PMC4957775

[ref12] Liu R. Han Q. Lin G. Mu S. Liu S. Yao S. . (2025). Analysis of the alkaline resistance mechanism of *Halomonas alkalicola* CICC 11012s by proteomics and metabolomics. Curr. Microbiol. 82:135. doi: 10.1007/s00284-024-04056-2 39945829

[ref13] Lu H. Guo S. Yang Y. Zhao Z. Xie Q. Wu Q. . (2025). Bikaverin as a molecular weapon: enhancing *Fusarium oxysporum* pathogenicity in bananas via rhizosphere microbiome manipulation. Microbiome 13:107. doi: 10.1186/s40168-025-02109-7, 40301992 PMC12042607

[ref14] Meinzner M. Ahmad N. Nielsen B. L. (2023). Halophilic plant-associated bacteria with plant-growth-promoting potential. Microorganisms 11:2928. doi: 10.3390/microorganisms11122910 38138054 PMC10745547

[ref15] Mon Y. Y. Bidabadi S. S. Oo K. S. Zheng S.-J. (2021). The antagonistic mechanism of rhizosphere microbes and endophytes on the interaction between banana and *Fusarium oxysporum* f. sp. *cubense*. Physiol. Mol. Plant Pathol. 116:101664. doi: 10.1016/j.pmpp.2021.101733

[ref16] Mukherjee P. Mitra A. Roy M. (2019). *Halomonas* rhizobacteria of *Avicennia marina* of Indian Sundarbans promote rice growth under saline and heavy-metal stresses through exopolysaccharide production. Front. Microbiol. 10:1207. doi: 10.3389/fmicb.2019.01207, 31191507 PMC6549542

[ref17] Nakkeeran S. Rajamanickam S. Saravanan R. Vanthana M. Soorianathasundaram K. (2021). Bacterial endophytome-mediated resistance in banana for the management of Fusarium wilt. 3 Biotech 11:267. doi: 10.1007/s13205-021-02833-5 PMC8124033 34017673

[ref18] Philippot L. Raaijmakers Jos M. Lemanceau P. van der Putten W. H. (2013). Going back to the roots: the microbial ecology of the rhizosphere. Nat. Rev. Microbiol. 11, 789–799. doi: 10.1038/nrmicro3109 24056930

[ref19] Platonovskiy N. G. Ibrasheva L. R. Obukhova N. I. Puchkova O. S. Babkina A. V. (2024). “International Banana trade: volumes, countries, and trends” in Sustainable development of the agrarian economy based on digital technologies and smart innovations. eds. Popkova E. G. Bogoviz A. V. Sergi B. S. (Cham: Springer Nature Switzerland), 25–30.

[ref20] Ploetz Randy C. (2015). Management of Fusarium wilt of banana: a review with special reference to tropical race 4. Crop Prot. 73, 7–15. doi: 10.1016/j.cropro.2015.01.007

[ref21] Posada L. F. Arteaga-Figueroa L. A. Adarve-Rengifo I. Cadavid M. Zapata S. Álvarez J. C. (2024). Endophytic microbial diversity associated with commercial cultivar and crop-wild-relative banana varieties could provide clues for microbial community management. Microbiol. Res. 287:127862. doi: 10.1016/j.micres.2024.127862 39121704

[ref22] Santoyo G. Moreno-Hagelsieb G. Orozco-Mosqueda M. C. Glick B. R. (2016). Plant growth-promoting bacterial endophytes. Microbiol. Res. 183, 92–99. doi: 10.1016/j.micres.2015.11.008 26805622

[ref23] Shao M.-W. Chen H.-J. Huang A.-Q. Zheng L. Li C.-j. Qin D. . (2025). Modulation of rhizosphere microbiota by *Bacillus subtilis* R31 enhances long-term suppression of banana Fusarium wilt. iMetaOmics 2:e70006. doi: 10.1002/imo2.70006 41675166 PMC12806485

[ref24] Thomas P. Sekhar A. C. (2014). Live-cell imaging reveals extensive intracellular cytoplasmic colonization of banana by normally non-cultivable endophytic bacteria. AoB Plants 6:plu002. doi: 10.1093/aobpla/plu002 24790123 PMC4038436

[ref25] Wang M. Zhou D. Jing T. Hu Y. Gao Z. Xie Q. . (2014). Endophyte isolation and broad-spectrum antagonistic bacteria screening from banana. Biotechnol. Bull. 8, 138–145. doi: 10.13560/j.cnki.biotech.bull.1985.2014.08.029

[ref26] Zhao M. Su Z. Long F. Zou Y. Mo T. Huang X. . (2024). Analysis of changes in rhizosphere soil microbial community structure of banana varieties resistant to wilt disease under continuous cropping. J. South. Agric. 55, 1–12. doi: 10.3969/j.issn.2095-1191.2024.01.001

